# CD147/EMMPRIN overexpression and prognosis in cancer: A systematic review and meta-analysis

**DOI:** 10.1038/srep32804

**Published:** 2016-09-09

**Authors:** Xiaoyan Xin, Xianqin Zeng, Huajian Gu, Min Li, Huaming Tan, Zhishan Jin, Teng Hua, Rui Shi, Hongbo Wang

**Affiliations:** 1 Department of Gynecology and Obstetrics, Union Hospital, Tongji Medical College, Huazhong University of Science and Technology, No. 1277, Jiefang Avenue, Jiangan District, 430022 Wuhan P. R. China; 2 Department of Pathophysiology, School of Basic Medicine, Tongji Medical College, Huazhong University of Science and Technology, No. 13, Hangkong Road, Qiaokou District, 430030 Wuhan P. R. China; 3 Department of General Surgery, Affiliated Hospital of Guizhou Medical College, No. 28, Guiyi Street, Yunyan District, 550000 Guiyang P. R. China; 4 Department of Gynaecology, Shanghai Clinical Center of Chinese Academy of Science, Shanghai, 100864 P. R. China; 5 Department of Gynecology and Obstetrics, Shenzhen Tumor Hospital, No. 113, Baohe Avenue, Longgang District, 518116 Shenzhen, P. R. China

## Abstract

CD147/EMMPRIN (extracellular matrix metalloproteinase inducer) plays an important role in tumor progression and a number of studies have suggested that it is an indicator of tumor prognosis. This current meta-analysis systematically reevaluated the predictive potential of CD147/EMMPRIN in various cancers. We searched PubMed and Embase databases to screen the literature. Fixed-effect and random-effect meta-analytical techniques were used to correlate CD147 expression with outcome measures. A total of 53 studies that included 68 datasets were eligible for inclusion in the final analysis. We found a significant association between CD147/EMMPRIN overexpression and adverse tumor outcomes, such as overall survival, disease-specific survival, progression-free survival, metastasis-free survival or recurrence-free survival, irrespective of the model analysis. In addition, CD147/EMMPRIN overexpression predicted a high risk for chemotherapy drugs resistance. CD147/EMMPRIN is a central player in tumor progression and predicts a poor prognosis, including in patients who have received chemo-radiotherapy. Our results provide the evidence that CD147/EMMPRIN could be a potential therapeutic target for cancers.

The incidence of various cancers has been increasing and cancer is the most deadly disease threatening human life. One of the main causes of deaths is the inherent metastatic property of malignant tumors. This poses great difficulty in developing clinical therapeutics. The multi-linked pathological processes of tumor metastasis include: basement membrane degradation, matrix permeability, forward movement of tumor cells including secondary growth, and interaction between tumor cells and host stromal cells. CD147/EMMPRIN, also known as basigin or M6 antigen, has been shown to play an important role in tumor metastasis by stimulating tumor stromal cells to produce matrix metalloproteinases (MMPs) and degrading basement membrane and stroma^1^.

CD147/EMMPRIN is a member of the immunoglobulin family and is widely expressed in a variety of human tissues and cells^1^. CD147/EMMPRIN functions to: (1) facilitate secretion of MMP-1, MMP-3, MMP-9 and membrane-type 1-MMP from cancer cells, fibroblasts and endometrial cells, leading to degradation of basement membrane and extracellular matrix, thus promoting tumor proliferation, invasion and metastasis^1^,^2^; (2) drive tumor angiogenesis by enhancing MMPs and vascular endothelia growth factor (VEGF) levels in cancer cells and the mesenchyme^3^; (3) regulate expression and activity of monocarboxylate transporters-1 (MCT-1) and MCT-4, and form complexes on the membrane to transport lactic acid produced by anaerobic glycolysis^4^; (4) develop chemoresistance in many cancers, probably by mediating activation of phosphatidylinositol 3-hydroxy kinase (PI3K) and mitogen-activated protein kinase (MAPK) pathways^5^,^6^,^7^,^8^; and (5) interact with α3β1, α6β1 integrins to regulate adhesion with extracellular matrix proteins, collagen, laminin or fibronectin and also promote expression of cyclophilin A to induce cancer cell proliferation^9^. All of these functions are regulated by CD147/EMMPRIN and are summarized in Fig. 1. The outcomes of many of the pathways regulated by CD147 have been associated with the adverse outcomes highlighted in our study.

It is highly upregulated in several malignant tumors^10^,^11^,^12^,^13^,^14^,^15^,^16^,^17^. A few recent studies^10^,^15^,^16^,^17^ have revealed a conflicting correlation between CD147/EMMPRIN and various outcomes in different cancers. A meta-analysis of the literature published previously suggests that elevated MCT-4 and CD147 expressions are associated with worse prognosis across many cancer types focusing on the aspect of tumor metabolism while the existing evidence lacks statistical power to draw a convincing conclusion^18^. The objective of this updated study was to systematically assemble all the existing CD147 literature, link the data to variable outcomes, perform a comprehensive meta-analysis to predict potential prognostic effects in different cancers, and provide further evidence to establish CD147/EMMPRIN as a key player in tumor progression from a number of perspectives.

## Results

**Figure 2:**
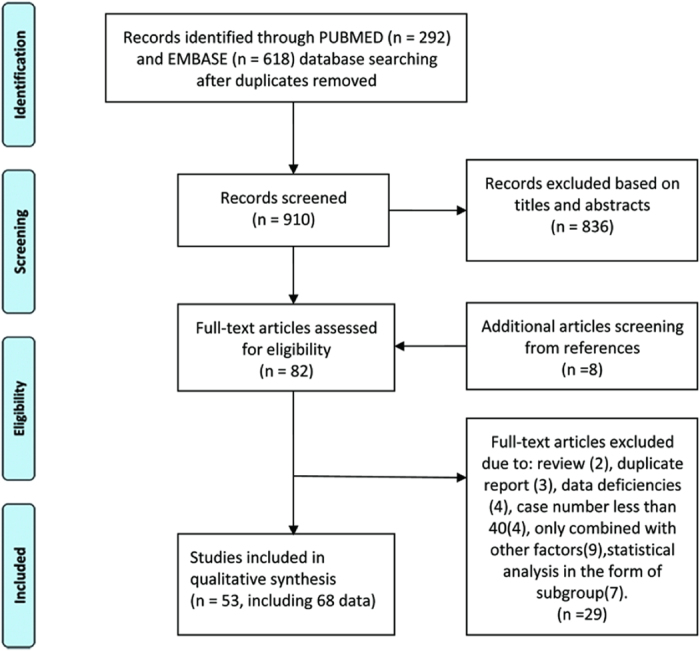


Our systematic literature search of CD147 and its correlation with different outcomes identified 910 articles. Among these, 836 articles were excluded and only 82 studies satisfied the inclusion eligibility for the meta-analysis. Upon further review of the full text articles, eight additional articles from reference sources were included. An additional 29 studies were excluded due to the following reasons: two articles were reviews; three were duplicate reports; four had insufficient data; four included only a few cases; nine explored the prognostic value of CD147/EMMPRIN in combination with other biomarkers, such as VEGF, MMP-2, CD44s, MCT-1; and seven studies were determined to be too complicated for subgroup analysis. The remaining 53 studies^5^,^6^,^7^,^9^,^10^,^11^,^12^,^13^,^14^,^15^,^16^,^17^,^19^,^20^,^21^,^22^,^23^,^24^,^25^,^26^,^27^,^28^,^29^,^30^,^31^,^32^,^33^,^34^,^35^,^36^,^37^,^38^,^39^,^40^,^41^,^42^,^43^,^44^,^45^,^46^,^47^,^48^,^49^,^50^,^51^,^52^,^53^,^54^,^55^,^56^,^57^,^58^,^59^ containing 68 datasets that met our inclusion criteria were included in this review (Fig. 2). Among the eligible studies, 33 provided survival information about the correlation of CD147/EMMPRIN expression with tumor prognosis using a multivariate model and 20 presented the same information using a univariate model. The characteristics of these two models are summarized in Tables 1 and 2, respectively.

The 53 eligible studies represented 26 different carcinomas or sarcomas and a median number of 204.5 patients (range, 40–600). CD147/EMMPRIN expression in these studies was mainly detected by immunohistochemistry (IHC). One publication had three groups of subjects from different cancers and was therefore considered as three different studies^49^. Ten publications^12^,^13^,^14^,^16^,^25^,^29^,^38^,^39^,^51^,^52^ presented two different prognostic results, while one publication^31^ presented three prognostic results. Some studies also investigated the link between CD147/EMMPRIN and chemotherapy^5^,^6^,^7^ or radiation resistance^57^. In terms of clinicopathological variables, most of the studies suggested that increased CD147/EMMPRIN expression correlated significantly with higher clinical grade, tumor size, invasion depth, lymphatic invasion, histological grade, and some additional parameters (Table 3).

### CD147/EMMPRIN and overall survival (OS)

**Figure 3:**
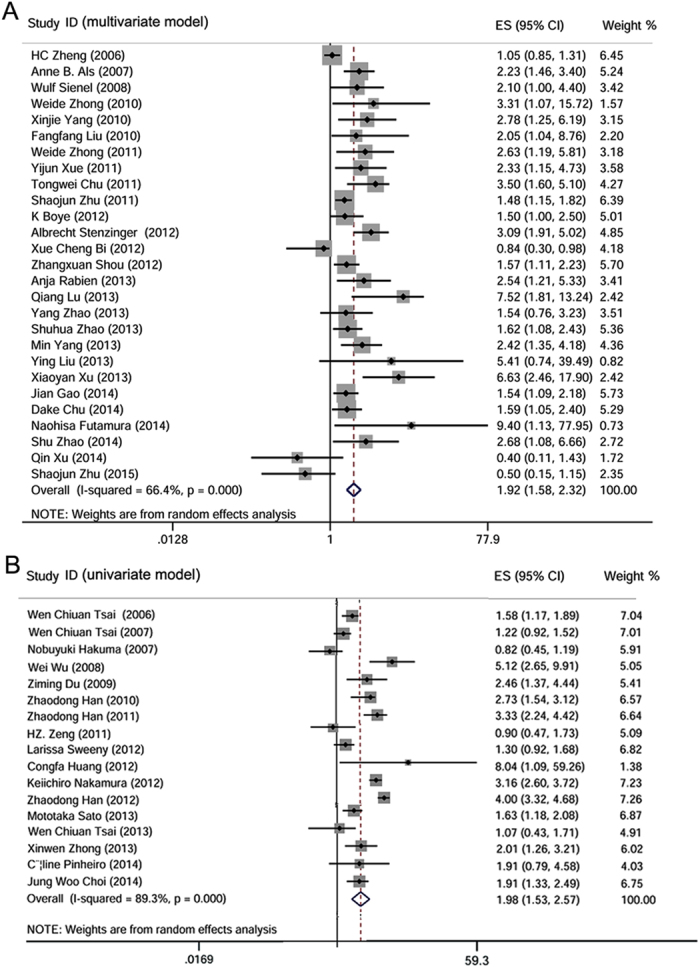
Qualitative meta-analysis of the association between CD147/EMMPRIN over-expression and overall survival (OS) in cancer patients. Panel A, represents the association of CD147/EMMPRIN positive expression with worse OS in multivariate model, while panel B, represents similar association by univariate model.

The 44 studies, with data from a total of 5,813 patients, showed that CD147/EMMPRIN expression was associated with worse OS, both in multivariate (meta-hazard ratio (HR) = 1.92; 95% confidence interval (CI): 1.58–2.32) and in univariate models (meta-HR = 1.98; 95% CI: 1.53–2.57) (Table 4; Fig. 3A,B). In addition, separate analysis of solid and non-solid tumors suggested that the OS of solid tumors was significantly associated with CD147 in both multivariate (meta-HR = 1.73; 95% CI: 1.44–2.08) and univariate (meta-HR = 2.06; 95% CI: 1.57–2.70) models. In contrast, OS of non-solid tumors only displayed significant association in the multivariate model (meta-HR = 3.72; 95% CI: 2.23–6.22), while there was no significant association in the univariate model analysis (meta-HR = 1.34, 95% CI: 0.77–2.32) (Table 4).

Furthermore, the subgroup analysis of tumors stratified by cancer type also demonstrated a significant association between higher CD147/EMMPRIN expression and adverse outcome of OS for breast carcinoma (meta-HR = 2.92; 95% CI: 1.85–4.60), bladder cancer (meta-HR = 2.32; 95% CI: 1.63–3.29), colorectal cancer (meta-HR = 2.14; 95% CI: 1.38–4.26), ovarian cancer (meta-HR = 1.57; 95% CI: 1.23–2.01) and osteosarcoma (meta-HR = 7.83; 95% CI: 3.18–19.27) in the multivariate model. However, an association was only observed in renal cell carcinoma (meta-HR = 1.87; 95% CI: 1.37–2.56) and bladder carcinoma (meta-HR = 2.51; 95% CI: 1.46–4.33), using the univariate model. In contrast, there was no association in gastric carcinoma (meta-HR = 1.33; 95% CI: 0.99–1.80) and lung cancer (meta-HR = 1.16; 95% CI: 0.63–2.12) (Table 4). We also found a similar association of CD147 with the adverse outcome (OS) in many other cancers, such as adenoid cystic carcinoma of salivary glands, esophageal squamous cell carcinoma, prostate cancer, pediatric medulloblastoma, glioblastoma, gallbladder carcinoma, nasopharyngeal carcinoma, tongue squamous cell carcinoma, endometrial cancer, uterine cervical carcinoma, hepatocellular carcinoma, pancreatobiliary adenocarcinoma, cutaneous squamous cell carcinoma, astrocytomas and soft tissue sarcomas (Supplementary Figure 1A,B).

### CD147/EMMPRIN and progression free survival (PFS), metastasis-free survival (MFS) and recurrence-free survival (RFS)

**Figure 4:**
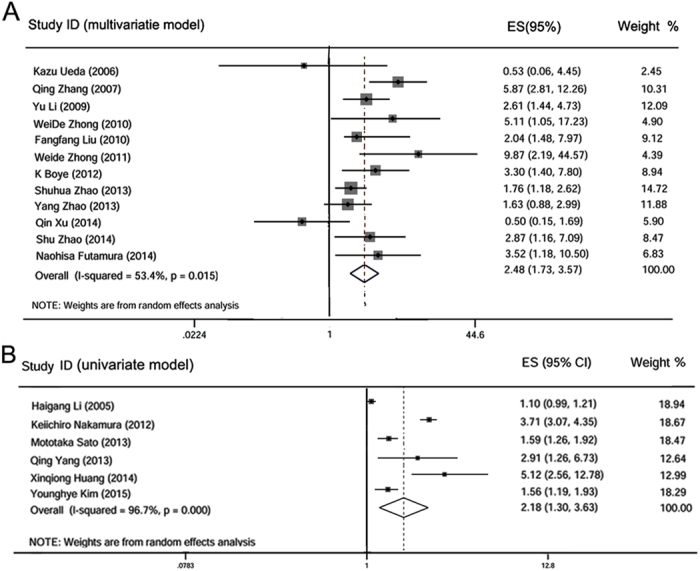
Qualitative meta-analysis of the association between CD147/EMMPRIN over-expression and PFS/MFS/RFS in cancer patients. Panel (A) represents the overall association of CD147/EMMPRIN positive expression with worse PFS/MFS/RFS in multivariate model; panel (B) depicts similar association in univariate model.

Analysis of 19 studies, with a total of 2,472 patients, showed that CD147/EMMPRIN expression was associated with worse PFS/MFS/RFS, both in multivariate (meta-HR = 2.32; 95% CI: 1.67–3.21) and univariate (meta-HR = 2.18; 95% CI: 1.30–3.63) models (Table 3 and Fig. 4A,B). Moreover, the HR estimates derived specifically from the solid tumors by both models showed a similar pattern of association (multivariate: meta-HR = 2.26; 95% CI: 1.61–3.18; univariate: meta-HR = 2.18; 95% CI: 1.30–3.63). Exclusive analysis of non-solid tumors by a multivariate model suggested a significant association (meta-HR = 3.52; 95% CI: 1.18–10.5) (Table 4).

Similarly, the subgroup analysis of tumors stratified by cancer type again demonstrated a significant association between CD147/EMMPRIN overexpression and adverse outcome of PFS/MFS/RFS in breast carcinoma (meta-HR = 2.50; 95% CI: 1.63–3.83) and ovarian cancer (meta-HR = 1.72; 95% CI: 1.23–2.40) in the multivariate model and renal cell carcinoma (meta-HR = 1.58; 95% CI: 1.34–1.85) in the univariate model. Similar associations were observed in hepatocellular carcinoma, bladder cancer, prostate cancer, colorectal cancer, osteosarcoma, gastric cancer and hypopharyngeal squamous cell carcinoma. However, we did not observe an association in cases of endometrial carcinoma, and the results were not consistent for cervical carcinoma (Supplementary Figure 2A,B).

### CD147/EMMPRIN and disease-specific survival (DSS)

**Figure 5:**
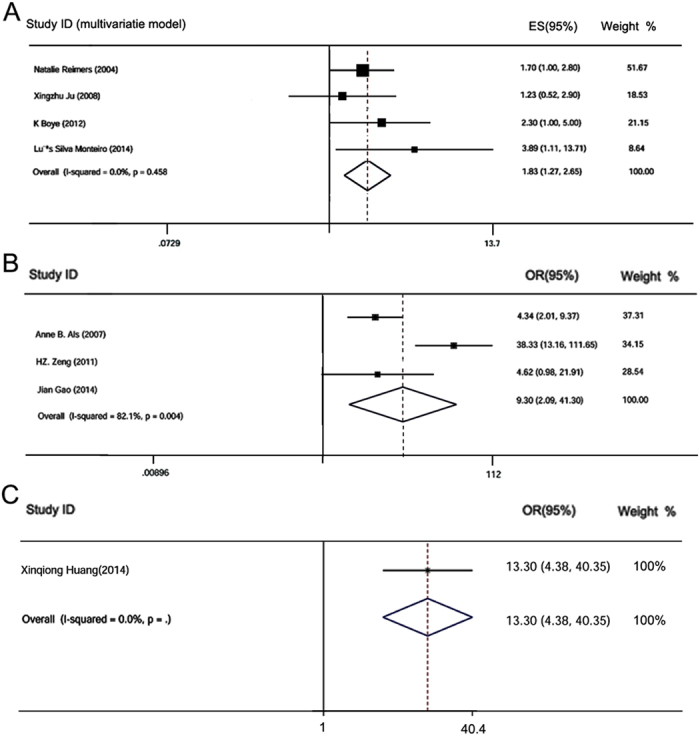
Qualitative meta-analysis of the association between CD147/EMMPRIN over-expression and disease free survival (DSS) in cancer patients, and to predict easier recurrence of drug resistance. Panel A, represents the association of CD147/EMMPRIN positive expression with worse DSS in multivariate model. Panels B, represents the potential of CD147/EMMPRIN positive expression to predict easier recurrence of drug resistance.

Quantitative analysis of five different studies, representing 1,031 patients, linked CD147/EMMPRIN overexpression with DSS in four different solid tumors, namely breast cancer^2^, colorectal cancer [24, 41], oral squamous cell carcinomas^7^ and cervical cancer^16^. Multivariate model analysis established that CD147/EMMPRIN expression was associated with a worse DSS (meta-HR = 1.83; 95% CI: 1.27–2.65) (Table 4; Fig. 5A). Similarly, univariate model analysis indicated that CD147 expression associated with worse DSS (meta-HR = 5.81; 95% CI: 4.16–7.46) (Table 4). This pattern was unchanged even in the subgroup analyses stratified by cancer type. Multivariate model analysis also identified the following associations with DSS: breast carcinoma (meta-HR = 1.70; 95% CI: 1.02–2.84), oral squamous cell carcinoma (meta-HR = 3.89; 95% CI: 1.11–13.71) and colorectal cancer (meta-HR = 2.30; 95% CI: 1.03–5.14). The only cancer for which we did not observe an association was uterine cervical carcinoma (meta-HR =1.23; 95% CI: 0.52–2.90) (Supplementary Figure 4).

### CD147/EMMPRIN and chemotherapy drug/radiation resistance

Among the included articles, three studies^5^,^6^,^7^ reported risk for CD147/EMMPRIN overexpression and chemotherapy drug resistance, while only one study^57^ reported radiation resistance. Quantitative analysis of three articles revealed that positive expression of CD147/EMMPRIN predicted recurrence of drug resistance (meta-OR = 9.30; 95% CI: 2.09–41.30) (Fig. 5B). In addition, CD147/EMMPRIN overexpression appeared to be linked to high risk of radiation resistance (OR = 13.30; 95% CI: 4.38–40.35) in cervical squamous cell carcinomas, although this is just based on one study (Fig. 5C).

### Heterogeneity analysis

There was evidence of significant heterogeneity (I^2^ > 50%) between OS and PFS studies, but not among DSS studies (Table 4). Therefore, the random-effect model was used in all analyses except for the DSS. For the OS and PFS studies, we conducted a meta-regression analysis using publication year, cancer type, sample size, and country as covariates. All covariates were entered into the meta-regression model simultaneously, and the covariates with the highest p values were omitted one at a time to identify sources of heterogeneity. The meta-regression did not identify any of these covariates as a significant source of heterogeneity for OS and PFS studies in multivariate, and indicated that cancer type may be the source of heterogeneity for OS studies in univariate analysis (Coef = 0.093, p = 0.042) (Supplementary Figure 4). Meta-regression was not used for univariate analysis of the PFS since these studies numbered less than 10.

### Sensitivity analysis

We also performed a sensitivity analysis to evaluate the effects of individual studies on the pooled HR estimates by omitting one study at a time. The HR estimates for the DSS and PFS/MFS/RFS in the multivariate model were not altered. However, the HR estimates for the OS in the multivariate model, and OS and PFS/MFS/RFS in the univariate model were altered when one, one and two studies were excluded, respectively (data not shown).

### Publication bias

**Figure 6:**
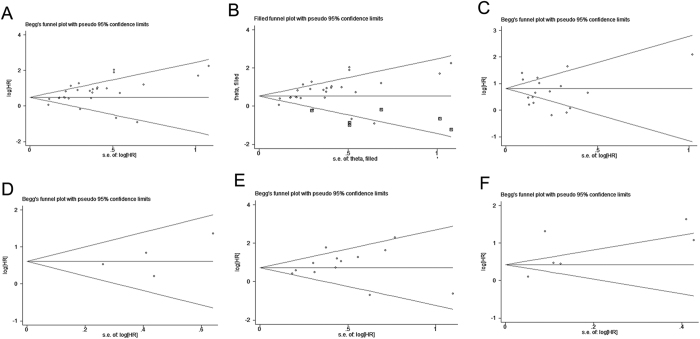
Assessment of publication bias for OS, DSS, and PFS/MFS/RFS studies. Panel (A) depicts the assessment of publication bias for multivariate model studies, by funnel plot analysis, whereas panel (B) shows funnel plot analysis using nonparametric Trim and Fill method. Panel (C) represents the publication bias in univariate model studies. Panel (D) represents the assessment of publication bias for multivariate model. Panels (E,F) represents assessment of publication bias for multivariate and univariate model studies, respectively.

To assess confidence in our study, we performed a publication bias analysis using the funnel plot and Egger’s and Begg’s rank correlation tests. There was no significant publication bias in both models for the DSS and PFS/MFS/RFS groups (Table 4; Fig. 6D–F). In the case of the OS group, the univariate model (Table 4; Fig. 6C) suggested no publication bias, but in the multivariate model, results were inconsistent based on the p value obtained by Begg’s rank test (0.05) and Egger’s test (0.007) (Table 4; Fig. 6A). These test results were nonparametric and therefore the Trim and Fill method was used to further verify this analysis. After filling the deleted studies (square dots), we found no obvious asymmetry in the funnel plot (Fig. 6B). Thus, the HR estimates for the prognostic value of CD147/EMMPRIN were not notably altered (data not shown). This suggests that there was no publication bias even in the OS group in the multivariate model.

## Discussion

CD147/EMMPRIN is a glycosylated, multifunctional molecule that participates in tumor progression^61^. In the present study, we quantitatively analyzed the data from 53 studies, including 68 datasets, to examine the associations between CD147/EMMPRIN expression and its prognosis predictive value in cancer. Previous studies have suggested that combination of CD147/EMMPRIN with other factors, especially VEGF^60^,^62^,^63^ and MMP-2^64^,^65^,^66^, can predict the prognosis of some cancers. Here, we exclusively studied the role of CD147/EMMPRIN in tumor prognosis. Our meta-analysis revealed that the prognosis in three adverse outcomes was significantly poor in cases with CD147/EMMPRIN overexpression. This was further confirmed in subgroup analyses of tumors stratified by cancer type. Meanwhile, the predictive role of CD147/EMMPRIN in cervical cancer and hepatocellular carcinoma prognosis has been controversial and its result in cervical carcinoma, endometrial carcinoma, pancreatobiliary adenocarcinoma and some additional tumors did not consistently reach significance. However, the sample size and studies in our stratified analysis were small, and our findings should be further verified.

CD147/EMMPRIN has been shown to be involved in the regulation of tumor cell invasion, metastasis, angiogenesis, anti-apoptosis, adhesion and facilitation of drug resistance through its association with various proteins^1^, such as MMP-2^19^, MMP-9^20^, Ki-67^14^, VEGF^19^, microvessel density^19^, C-erbB-2^12^, S100A4^31^, a disintegrin and metalloproteinase 17^32^, lewis y antigen^6^, fascin^43^, caveolin-1^48^, hypoxia inducible factor 1 alpha^9^, cyclophilin A^9^, CD44v6^54^, cyclooxygenase-2^54^, receptor for activated C kinase 1^55^ and metabolism related factors like MCT-1^56^, MCT-4^56^, and glucose transporter -1^57^. However, there is some conflicting literature refuting associations with paxillin^42^, syndecan-1^42^, and MMP-2 and MMP-9^22^. In addition, CD147 expression has been proposed to inversely correlate with estrogen and progesterone expression^10^,^12^. Furthermore, CD147/EMMPRIN was positively associated with clinicopathological variables in most studies (Table 3). It is speculated that CD147/EMMPRIN interacting with many proteins representing various molecular or biological pathways contributes to malignant progression, eventually causes adverse clinical outcomes.

Previous meta-analyses didn’t explore any significant association between CD147/EMMPRIN and susceptibility of radio-chemotherapy. In the view of more studies are warranted to validate CD147/EMMPRIN association with chemotherapy and radiation resistance prediction^5^,^6^,^7^,^57^. It has been suggested that chemotherapy drugs combined with CD147-targeted therapy may increase the sensitivity of tumor cells to several different chemotherapeutics, and can result in more effective inhibition of tumor proliferation and recurrence^7^,^67^. Our study also established that increased CD147/EMMPRIN expression is linked to high risk of drug resistance and our preliminary analysis linked CD147 to radiation resistance. It follows that CD147/EMMPRIN has been proposed to be an important potential therapeutic target.

The results of the present study must be interpreted with caution due to the presence of substantial heterogeneity. In addition, there were several limitations in this study. First, the composition of the cancer type or stage varied between studies, and the detection and corresponding cut-offs varied. For instance, some studies only included pediatric patients^26^ while others included older patients^10^. Also, in non-small-cell lung cancer, CD147/EMMPRIN was associated with poor survival in patients with adenocarcinoma only, but not with squamous cell carcinoma^22^. The scoring criteria were also inconsistent. Second, the follow-up time varied across studies, which may have contributed to the non-homogeneity of prognostic information. Sensitivity analysis results also showed instability within individual articles. Third, this study was based on published articles only and, since negative data are hard to publish, there could be publication bias, which is an inherent limitation of all meta-analyses, irrespective of outcomes from the Egger’s linear regression test and Begg’s rank correlation test.

## Conclusion

In summary, this meta-analysis indicated that higher expression of CD147/EMMPRIN potentially may be a prognostic marker for most cancers, and thus can serve as a potential therapeutic target. We further verified that CD147/EMMPRIN had a complex role in tumor progression by crosstalk with numerous factors. However, additional multicenter prospective studies are warranted to confirm these findings, especially in various types of tumors.

## Methods

### Study identification

We searched PubMed and Embase databases through March 2015 to identify relevant studies for inclusion in our meta-analysis. The following keywords were used in the literature searches; “CD147”, “extracellular matrix metalloproteinase inducer”, “EMMPRIN”, “basigin”, “survival”, “prognosis”, “tumor”, “cancer”, “carcinoma”, “neoplasm”, or their combinations. Eligible articles were selected based on title, abstract and full text. If the same patient cohort was reported in multiple publications, only the most complete and most recent publication was selected. We also searched of the reference lists from electronically identified articles.

### Inclusion and exclusion criteria

Based on the Reporting Recommendations for Tumor Marker Prognostic Studies (REMARK) guidelines, we included studies that met all of the following criteria: (1) written in English; (2) reported quantitative outcomes from prognostic association studies of tumor and CD147/EMMPRIN; (3) described outcomes as overall survival (OS), disease-specific survival (DSS), progression-free survival (PFS), metastasis-free survival (MFS) or recurrence-free survival (RFS), depending upon the study; (4) had a minimum of 40 case numbers describing prognosis; (5) provided a detailed protocol, including the source of raw materials, methodology, quantification methods, and scoring criteria; (6) precisely defined the time-to-event outcome, time to follow-up, and the median follow-up time; and (8) data were presented as the estimated hazard ratios (HRs) with 95% confidence intervals (CIs) or in the case where HRs or 95% CIs were not reported directly, a calculation was used to determine if the conditions of the study were suitable for inclusion^68^. Multivariate analyses were used for statistical analysis biomarkers that had independent prognostic factors for cancers after adjusting for one or more additional standard clinical prognostic variables like age, pathology, stage, grade, lymphatic metastasis or other biological marker variables. Studies were excluded if they were case reports, case-only studies, letters, reviews, reported insufficient data, lacked statistical analysis, combined with other factors, or were duplicate studies. All studies were independently reviewed by two authors, and in a case of conflict a third author resolved the issue after thorough discussion.

### Data collection and analysis

We assessed heterogeneity using the Chi^2^ test and I^2^ test^69^. If heterogeneity was present, meta-regression was used to determine the source. We combined data from different trials using a fixed-effect model when there was no significant heterogeneity in populations (I^2^ < 50%) and a random-effect model when there was considerable heterogeneity. If heterogeneity was present, meta-regression was used to determine the source. Variables were synthesized using HR/OR. By convention, an overall HR (OR) > 1 with a 95% CI implied a poor outcome (high risk) for the group with either positive or negative biomarker expression. The high HR value corresponded with poor survival. To evaluate the effects of individual studies on the pooled HR estimates, we performed a sensitivity analysis omitting one study at a time. The statistical significance was set at 0.05. We used funnel plot asymmetry using Egger’s linear regression test and Begg’s rank correlation test to assess the publication bias. If both test results were inconsistent, Nonparametric Trim and Fill method was used to verify the results^70^. A P value of <0.05 suggested significant publication bias. All statistical analyses were performed using STATA 12.0 (StataCorp, College Station, TX) statistical software.

## Additional Information

**How to cite this article**: Xin, X. *et al*. CD147/EMMPRIN overexpression and prognosis in cancer: A systematic review and meta-analysis. *Sci. Rep.* **6**, 32804; doi: 10.1038/srep32804 (2016).

## Supplementary Material

Supplementary Information

## Figures and Tables

**Figure 1:**
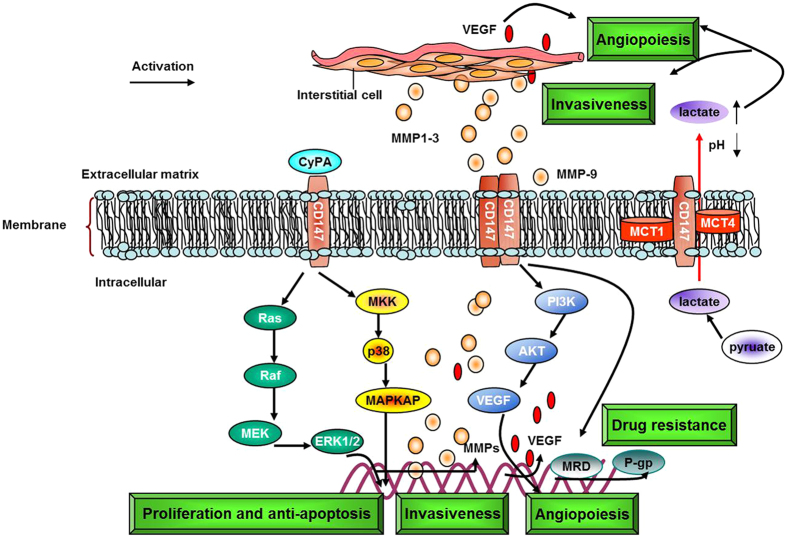


**Table 1:** Characteristics of studies exploring the relationship between CD147/EMMPRIN expression and tumor prognosis (Multivariate model).

Author	Year	Country	Cancer type	Stage/grade	Number Median (range)	Age	Follow-up time Median (range)	Detection method	Cut-off	Outcome
Natalie Reimers-10	2004	Germany	Breast carcinoma	pT1-pT4	600	>50y	63m (1–176m)	TMA/IHC	score ≥ 1	DSS
HC Zheng-19	2006	Japan	Gastric carcinoma	0–IV	219	66.8y (38–88y)	40.4m (0.2m–12.2y)	TMA/IHC	≥5%	OS
Kazu Ueda-20	2006	Japan	Endometrial carcinoma	I–IV	112	55.3 ± 11.7	NA	IHC	score > 2	RFS
Qing Zhang-21	2007	China	Hepatocellular carcinoma	I–IV	111	47.47 ± 9.55 (24–66y)	26m (30–1880d)	IHC	≥1%	RFS
Anne B. Als-5	2007	Denmark	Bladder Cancer	T4b, N2–3, M1	124	62.6y (31–78y)	56.5 (19.5–129.8) m	IHC	κ values of 0.83	OS
Wulf Sienel-22	2008	Germany	Non-small-cell lung cancer	T1–T4	57	60y (37–80y)	36 m (4–156)	IHC	score > 200	OS
Xingzhu Ju-23	2008	China	Cervical Cancer	Ib2–IIb	75	49.7y (21–72y)	52m (3–168m)	IHC	≥1%	DSS
Yu Li-11	2009	China	Breast carcinoma	infiltrating	106	NA	63.5m (7–170)	TMA/IHC	≥30%	RFS
Fangfang Liu-12	2010	China	Breast carcinoma	Invasive	186	52.5y (23–85y)	64.8m (7–170m)	IHC	≥1%	OS, PFS
Xinjie Yang-24	2010	China	Adenoid cystic carcinoma of salivary glands	I–IV	72	NA	76.76 ± 37.47 m; (9–178 m)	IHC	≥5%	OS
Wei-De Zhong-25	2010	China	Bladder cancer	T1–T4	101	68.1y (46–82y)	36m	IHC	≥5%	OS, PFS
Tongwei Chu-26	2011	China	Pediatric Medulloblastoma	M0–M4 (Metastatic stage)	56	Paediatric patients	5–y	IHC	≥5%	OS
Yijun Xue-27	2011	China	Bladder cancer	pT1–pT4	108	58.3y (31–82y)	35.5 m (3–86m)	TMA/IHC	≥1%	OS
Shaojun Zhu-28	2011	China	Esophageal Squamous Cell Carcinoma	NA	86	40–78y	4–6 y	IHC	≥5%	OS
Weide Zhong-29	2011	China	Prostate cancer	pT2–pT3	240	61.81 ± 6.54y/61.94 ± 5.83y	NA	TMA/IHC	≥5%	MFS, OS
Xuecheng Bi-30	2012	China	Human seminomas	pT1–pT4	65	21.66 ± 10.18y	5 y	IHC	≥5%	OS
K Boye-31	2012	Norway	Colorectal cancer	I–III	242	70y (21–98 y)	9.1y (8.2–10.0y)	IHC	≥5%	MFS, DSS, OS
Zhangxuan Shou-32	2012	China	Gastric Cancer	I–IV	436	64y (30–91y)	>5y	TMA/IHC	≥5%	OS
Albrecht Stenzinger -33	2012	Germany	Colorectal cancer	I–IV	285	66.6y	NA	TMA/IHC	score > 6	OS
Shuhua Zhao-13	2013	China	Ovarian cancer	I–IV	146	52.8y (26–79y)	36m (7–82m)	IHC	score ≥ 4	OS, PFS
Ying Liu-34	2013	China	Breast cancer	I–III	189	NA	NA	IHC	≥10%	OS
Anja Rabien-35	2013	Germany	Renal cell carcinoma	pN0/M0	181	60y (21–86y)	112m (0–194m)	TMA/IHC	score ≥ 1	OS
Xiaoyan Xu-36	2013	China	Non-small lung cancer	I–IV	136	60y (35–82y)	28m (1–87m)	IHC	≥1%	OS
Min Yang-37	2013	China	Glioblastoma	NA	206	53.6y (14–78y)	12.3 m (1–60 m)	IHC	score ≥ 1	OS
Yang Zhao-14	2013	China	Ovarian carcinomas	I–IV	88	51.2y (20–81y)	52m (1–103m)	TMA/IHC	score ≥ 1	RFS, OS
Qiang Lu-17	2013	China	Osteosarcoma	IIA–III	55	NA	32m (8–72m)	IHC	≥5%	OS
Dake Chu-38	2014	China	Gastric cancer	T1–T4	223	NA	41.8m (DFS)/58.0m (OS)	IHC	≥5%	OS, PFS
Qin Xu-39	2014	China	Cervical carcinoma	Ia1–IIb	110	NA	NA	IHC	≥10%	OS, PFS
Shu Zhao-40	2014	China	Breast cancer	I–III	127	49y (30–68y)	NA	IHC	≥10%	OS, PFS
Naohisa Futamura-16	2014	Japan	Osteosarcoma	IIA–IIB	53	20y (4–57y)	72m (8–200m)	IHC	score ≥ 1	OS, DFS
Jian Gao-43	2014	China	Ovarian cancer	I–IV	92	NA	NA	IHC	≥5%	OS
Luís Silva Monteiro-15	2014	Portugal	Oral squamous cell carcinomas	I–IV	74	62.3 ± 15.3y (25–96y)	36.45 ± 31.7m	TMA/IHC	score > 3	CSS
Shaojun Zhu-41	2015	China	Hepatocellular carcinoma	NA	50	31–76y	4y	IHC	≥5%	OS

**Table 2:** Characteristics of studies exploring the relationship between CD147/EMMPRIN expression and tumor prognosis (Univariate model) V.

Author	Year	Country	Cancer type	Stage/grade	Number	Age Median (range)	Follow-up time Median (range)	Detection method	Cut-off	Outcome
Hai-Gang Li-42	2005	China	Hepatocellular carcinoma	grade1–4	51	51 ± 11y (24–69y)	26m (5–90m)	IHC	≥10%	DFS
Wen-Chiuan Tsai -43	2006	China	Renal cell carcinoma	I–IV	100	NA	5–year	TMA/IHC	score > 130	OS
Nobuyuki Hakuma-44	2007	Japan	Non-Small Cell Lung Cancers	stage I	95	62.8 ± 9.1	5–y	IHC	score > 3	OS
Wen Chiuan Tsai-45	2007	China	Pancreatobiliary adenocarcinoma	T4	70	NA	2–y	IHC	score > 100	OS
Wei Wu-46	2008	China	Gallbladder carcinoma	I–IV	108	51.63 ± 10.08y (35–76y)	5–9y	IHC	≥5%	OS
Daniel Buergy-47	2009	Germany	Colorectal Cancer	I–IV	40	NA	30 months	IHC	≥5%	DSS
Ziming Du-48	2009	China	Nasopharyngeal carcinoma	I–IV	157	46y (14–86y)	5–y	TMA/IHC	≥5%	OS
Zhaodong Han-49	2010	China	Renal/Bladder/Prostate carcinoma	I–IV	52/58/101	57.26 ± 11.2y	1–5y	IHC	≥5%	OS
H. Z. Zeng-7	2011	China	Non-small-cell lung cancer	III/IV	118	NA	NA	IHC	≥25%	OS
Congfa Huang-9	2012	China	Tongue squamous cell carcinoma	I–IV	80	NA	69m (2–106m)	IHC	≥10%	OS
Larissa Sweeny-50	2012	USA	Cutaneous Squamous Cell Carcinoma	III–IV	56	72 ± 12 (42–91)	>2y	IHC	≥25%	OS
Keiichiro Nakamura-51	2012	Japan	Endometrial cancer	I–IV	134	57.7y (28–85)	NA	IHC	≥10%	DFS, OS
Mototaka Sato-52	2013	Japan	Renal cell carcinoma	I–IV	50	27–82 (62)	52 (1–114 m)	IHC	score ≥ 1	OS, PFS
Wen Chiuan Tsai-53	2013	China	Astrocytomas	III–IV	77	NA	NA	GDS1962 database	1,500	OS
Qing Yang-54	2013	China	Hypopharyngeal Squamous Cell Carcinoma	I–IV	80	60.73y (42–78)		IHC	≥P90 level	RFS
Xinwen Zhong-55	2013	China	Lung cancer	T1	180	60y (37–75y)	60m (3–96m)	IHC	≥25%	OS
Jung-Woo Choi-56	2014	Korea	Urothelial carcinoma of the bladder	Ta–T4	360	69y (23–97y)	36m	TMA/IHC	score > 16	OS
Xin-Qiong Huang-57	2014	China	Cervical squamous cell carcinoma	IB-IVA	132	51y (28–80y)	45m (2–85.5m)	IHC	≥5%	PFS
Céline Pinheiro-58	2014	Portugal	Soft tissue sarcomas	I–III	84	NA	NA	IHC	score ≥ 3	OS
Younghye Kim-59	2015	Korea	Clear cell renal cell carcinoma	I–IV	180	25–83y (58y)	40.7m (1–173)	TMA/IHC	score > 17	PFS

**Table 3:** Relationship between CD147/EMMPRIN overexpression and clinical and pathological factors.

Author	Cancer type	Relative to other factors	Relative to clinicopathologic variables
**Multivariate**			
Natalie Reimers-10	Breast carcinoma	ER, PR (inversely)	High tumor grade, histologically determined mitotic index, tumor size, inversely correlated to age
HC Zheng-19	Gastric carcinoma	ki-67, MMP-2, MMP-9, VEGF, MVD	Tumour size, depth of invasion, lymphatic invasion, not with lymph node metastasis, UICC staging or differentiation
Kazu Ueda-20	Endometrial carcinoma		Advanced stage, poorly differentiated carcinoma, lymph node metastasis, lymphatic vessel infiltration, pathological high risk group
Qing Zhang-21	Hepatocellular carcinoma	MVD-CD34; MMP-2 in pericancerous, not in cancerous lesions; VEGF	pTNM tumor stages, tumor size and venous invasion, IV stage and large tumor size, preoperative AFP level; not: viral hepatitis, the number of tumor nodules, lymph node metastasis
Anne B. Als-5	Bladder Cancer		Visceral metastases
Wulf Sienel-22	Non-small-cell lung cancer	MMP-2 (no), MMP-9 (no)	
Xingzhu Ju-23	Cervical Cancer		Pelvic lymph node metastasis, no correlation with clinical stage and histopathology
Yu Li-11	Breast carcinoma		
Fangfang Liu-12	Breast carcinoma	C-erbB-2; ER, PR (inversely)	Histological grade, local recurrence, distant metastasis and tumor mortality
Xinjie Yang-24	Adenoid cystic carcinoma of salivary glands	MMP-2, MMP-9, VEGF, Ki-67 index, MVD	Tumor size, histotypes, clinical stage, perneural invasion, vascular invasion, metastasis
Wei-De Zhong-25	Bladder cancer		Tumor stage and grade, status of carcinoma *in situ*, tumor recurrence, tumor progression
Tongwei Chu-26	Pediatric Medulloblastoma		Higher metastatic stage, aggressive histopathological type, necrosis, undifferentiated tumor
Yijun Xue-27	Bladder cancer		Lymph node status, tumor stage, histologic grade
Shaojun Zhu-28	Esophageal Squamous Cell Carcinoma		Lymph node metastasis cases, differentiation, depth of tumor invasion
Weide Zhong-29	Prostate cancer		Gleason score, positive surgical margin status, reduced PSA failure-free survival
Xuecheng Bi-30	Human seminomas	MMP-2	advanced T, N and M stage, poor differentiation types
K Boye-31	Colorectal cancer	S100A4	no associations with any of the clinical or histopathological parameters
Zhangxuan Shou-32	Gastric Cancer	ADAM17	Age, tumor size, location, depth of invasion, TNM stage, Lauren’s classification, vessel invasion, and lymph node and distant metastasis of tumor
Albrecht Stenzinger -33	Colorectal cancer		Trend correlation with stage, distant metastasis, blood vessel invasion, and Dukes classification
Shuhua Zhao-13	Ovarian cancer		Lymph-vascular space involvement, lymph node metastasis
Ying Liu-34	Breast cancer		
Anja Rabien-35	Renal cell carcinoma		pT stage and Fuhrman grading
Xiaoyan Xu-36	Non-small lung cancer		Tumor diameter, lymph node status, tumor stage
Min Yang-37	Glioblastoma		Karnofsky performance status (KPS) score
Yang Zhao-14	Ovarian carcinomas	Ki-67	FIGO staging, dedifferentiation
Qiang Lu-17	Osteosarcoma		Pathological classification, percentage of dead cells
Dake Chu-38	Gastric cancer		Invasion, metastasis and TNM stage
Qin Xu-39	Cervical carcinoma		FIGO clinical stage, lymph node metastasis, parametrium invasion, and differentiation
Shu Zhao-40	Breast cancer	MMP-9, Ki67	Lymph node metastasis, high pathological grade, tumor size larger than 2 cm
Naohisa Futamura-16	Osteosarcoma	MT1-MMP	Not associated with age, gender, anatomic location, necrosis after neoadjuvant chemotherapy, or surgical stage
Jian Gao-43	Ovarian cancer	Lewis y antigen	Drug-resistant
Luís Silva Monteiro-15	Oral squamous cell carcinomas	Ki-67	Advanced tumor stages, histological grade
Shaojun Zhu-41	Hepatocellular carcinoma		No relationship between differentiation, HBV infection, significantly opposite to cirrhosis
**Univariate**			
Hai-Gang Li-42	Hepatocellular carcinoma	paxillin and syndecan-1 (no)	Not associated with serum AFP level, HBsAg status, presence of microsatellite nodule, tumor size, presence of cirrhosis and necrosis, differentiation, presence of portal vein thrombosis and extra-hepatic metastasis
Wen-Chiuan Tsai -43	Renal cell carcinoma	fascin	Histological grades and clinical stages
Nobuyuki Hakuma-44	Non-Small Cell Lung Cancers		Well differentiated, not associated with any of the following variables: age, gender, histology, pT or pN classification, and pathological stage
Wen Chiuan Tsai-45	Pancreatobiliary adenocarcinoma	fascin	Histologic grades and clinical stages
Wei Wu-46	Gallbladder carcinoma	MMP-2	Nevin stages of tumor tissues, histological differentiated degree, distant metastasis
Daniel Buergy-47	Colorectal Cancer		pT or pN status, metastasis
Ziming Du-48	Nasopharyngeal carcinoma	Cav-1	Metastasis of the disease
Zhaodong Han-49	Renal/Bladder/Prostate carcinoma		TNM stages, histological subtypes
H. Z. Zeng-7	Non-small-cell lung cancer		No association (overall CD147); (membranous CD147) associated with a poor response to chemotherapy
Congfa Huang-9	Tongue squamous cell carcinoma	HIF-1a, VEGF-A, VEGF-C, CypA	Recurrence and node metastasis
Larissa Sweeny-50	Cutaneous Squamous Cell Carcinoma		Node positive disease
Keiichiro Nakamura-51	Endometrial cancer		FIGO stage, histology, depth of myometrial invasion, cervical involvement, lymph node metastasis, lymph vascular space involvement, peritoneal cytology
Mototaka Sato-52	Renal cell carcinoma	anti-CD34	Pathological T stage, clinical M stage, AJCC stage, Fuhrman grade, microvessel area of immature vessels
Wen Chiuan Tsai-53	Astrocytomas		WHO grades
Qing Yang-54	Hypopharyngeal Squamous Cell Carcinoma	CD44v6; COX-2	T classification, lymph node metastasis and clinical stage
Xinwen Zhong-55	Lung cancer	RACK1	Differentiation, Lymph node metastasis
Jung-Woo Choi-56	Urothelial carcinoma of the bladder	MCT1, MCT4	High World Health Organization grade, advanced tumor node metastatis stage, and nonpapillary growth type
Xin-Qiong Huang-57	Cervical squamous cell carcinoma	GLUT-1	Histopathological grade, Tumor diameter, radiation-resistant
Céline Pinheiro-58	Soft tissue sarcomas	MCT1, MCT4	Disease progression
Younghye Kim-59	Clear cell renal cell carcinoma		High grade, tumor necrosis, larger tumor size

**Table 4:** Meta-analysis of association between CD147/EMMPRIN expression and tumor prognosis.

	OS			PFS			DSS					
	No/case	meta-HR (95CI%) P*	I^2^ (P^#^)	No/case	meta-HR (95CI%) P*	I% (P^#^)	No/case	meta-HR (95CI%) P*	I^2^ (P^#^)			
**Multivariate**												
**Total**	27/3933	1.92 (1.58–2.32) 0.000	66.4 (0.000)	13/1845	2.32 (1.67–3.21) 0.000	56.3 (0.007)	4/991	1.83 (1.27–2.65) 0.001	0.00 (0.458)			
**Solid tumor**	23/3563	1.73 (1.44–2.08) 0.000	60.7 (0.000)	12/1792	2.26 (1.61–3.18) 0.000	58.5 (0.005)	4/991	1.83 (1.27–2.65) 0.001	0.00 (0.458)			
**Nun-solid tumor**	4/370	3.72 (2.23–6.22) 0.000	36.6 (0.192)	1/53	3.52 (1.18–10.5) 0.024		0					
Breast carcinoma	5/695	2.92 (1.85–4.60) 0.000	5.00 (0.378)	3/419	2.50 (1.63–3.83) 0.000	0.00 (0.846)						
Bladder cancer	3/333	2.32 (1.63–3.29) 0.000	0.00 (0.860)									
Gastric carcinoma	3/878	1.33 (0.99–1.80) 0.060	62.0 (0.072)									
Colorectal cancer	2/527	2.14 (1.38–4.26) 0.035	77.9 (0.033)									
Ovarian cancer	3/326	1.57 (1.23–2.01) 0.000	0.00 (0.982)	2/234	1.72 (1.23–2.40) 0.002	0.00 (0.833)						
Osteosarcoma	2/108	7.83 (3.18–19.27) 0.000	0.00 (0.852)									
**Total publish bias**	**0.05 (Begg’ test)**		**0.007 (Egger’ test)**	**0.300 (Begg’ test)**		**0.259 (Egger’ test)**	**0.734 (Begg’ test)**		**0.469 (Egger’ test)**			
**Univariate**												
Total	17/1880	1.98 (1.53–2.57) 0.000	89.3 (0.000)	6/627	2.18 (1.30–3.63) 0.003	96.7 (0.000)	1/40	5.81 (4.16–7.46) 0.037				
**Solid tumor**	15/1719	2.06 (1.57–2.70) 0.000	90.4 (0.000)	6/627	2.18 (1.30–3.63) 0.003	96.7 (0.000)	1/40	5.81 (4.16–7.46) 0.037				
Nun-solid tumor	2/161	1.34 (0.77–2.32) 0.300	2.90 (0.310)	0			0					
Renal cell carcinoma	3/202	1.87 (1.37–2.56) 0.000	70.9 (0.032)	2/230	1.58 (1.34–1.85) 0.000	0.00 (0.91)						
Lung Cancer	3/393	1.16 (0.63–2.12) 0.630	74.1 (0.021)	—			—					
Bladder carcinoma	2/418	2.51 (1.46–4.33) 0.001	81.9 (0.019)	—			—					
**Total publish bias**	**0.773 (Begg’ test)**		**0.228 (Egger’ test)**	**−0.707 (Begg’ test)**		**−0.304 (Egger’ test)**						

P* present P for HR, P# present P for I2.
