# Supplementary material for: CD147/EMMPRIN overexpression and prognosis in cancer: A systematic review and meta-analysis
Source: Sci Rep. 2016 Sep 9;6:32804. doi: 10.1038/srep32804 (PMC5016850; doi:10.1038/srep32804)

**Supplementary information**

**Title: CD147/EMMPRIN over-expression and prognosis in cancer: A systematic review and meta-analysis**

Xiaoyan Xin, Xianqin Zeng， Huajian Gu, Min Li, Huaming Tan, Zhishan Jin, Teng Hua, Rui Shi, Hongbo Wang.

**Supplementary Figure 1.**

Sub-group analysis of the association between CD147/EMMPRIN over-expression and cancer patients. Panels A-B represents sub-group analysis of CD147/EMMPRIN positive expression with OS in multivariate and univariate models, respectively.
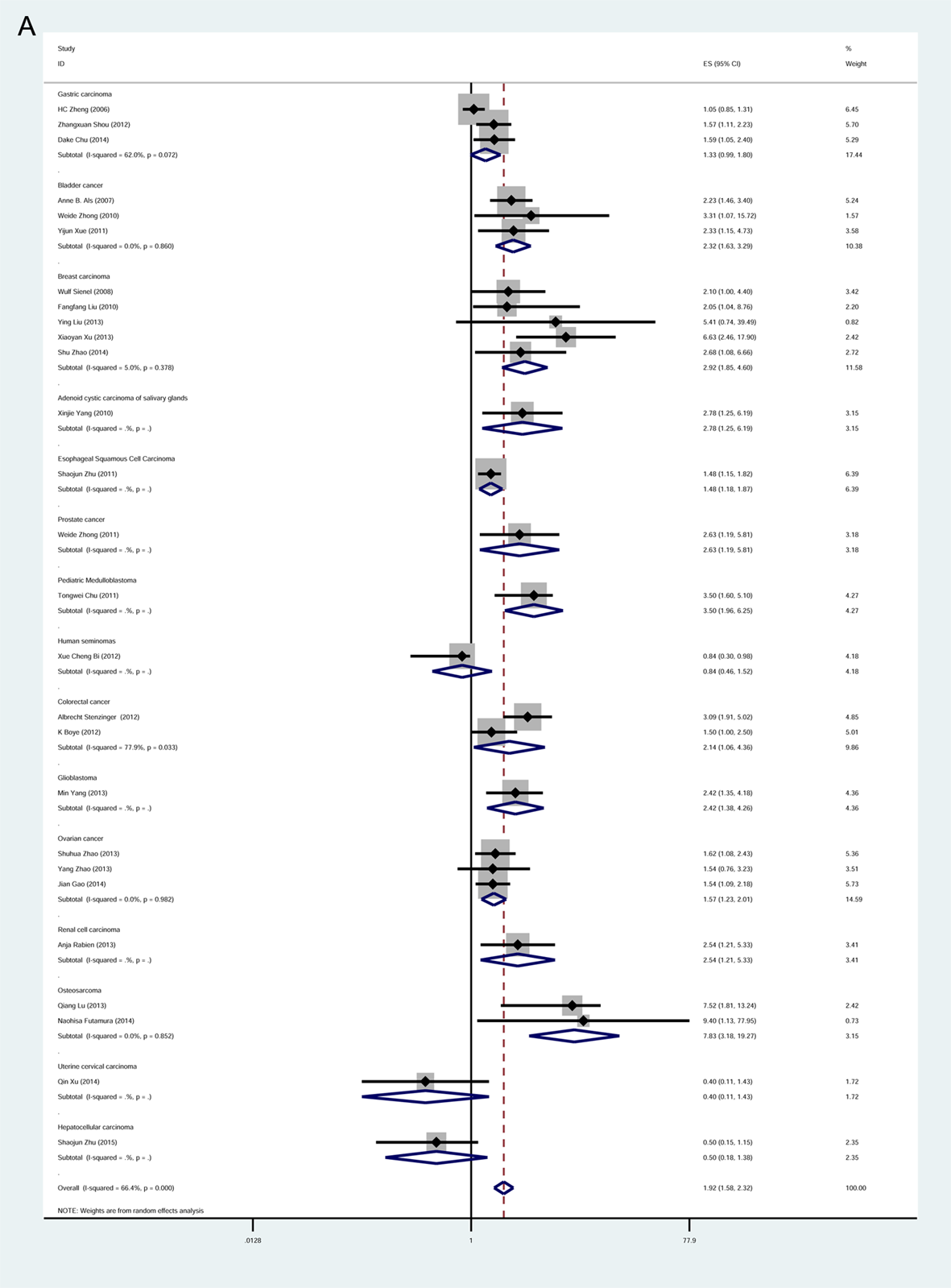

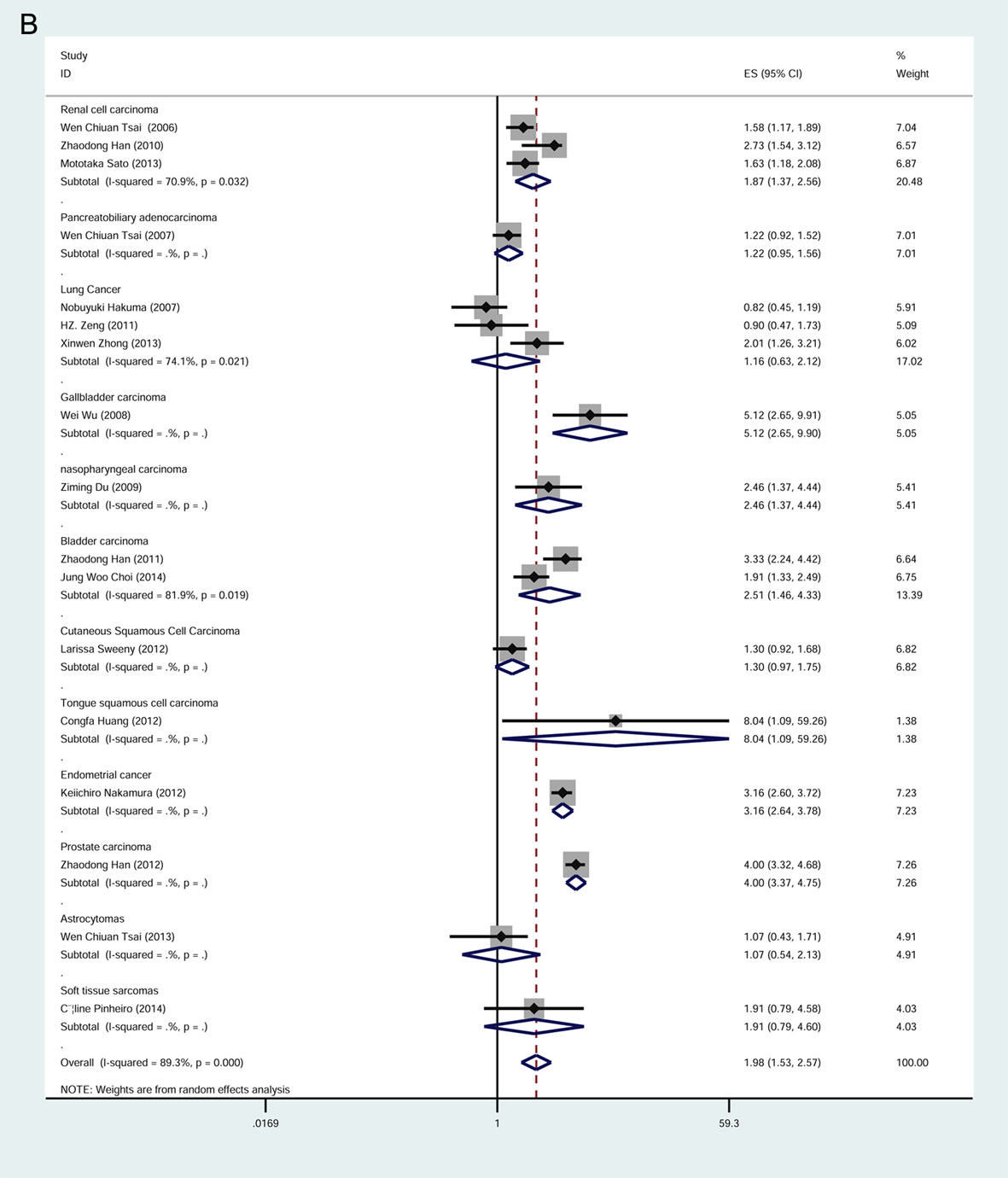


**Supplementary Figure 2.**

Sub-group analysis of the association between CD147/EMMPRIN over-expression and cancer patients. Panels A-B show similar analysis with PFS/MFS/RFS in multivariate and univariate models, respectively.


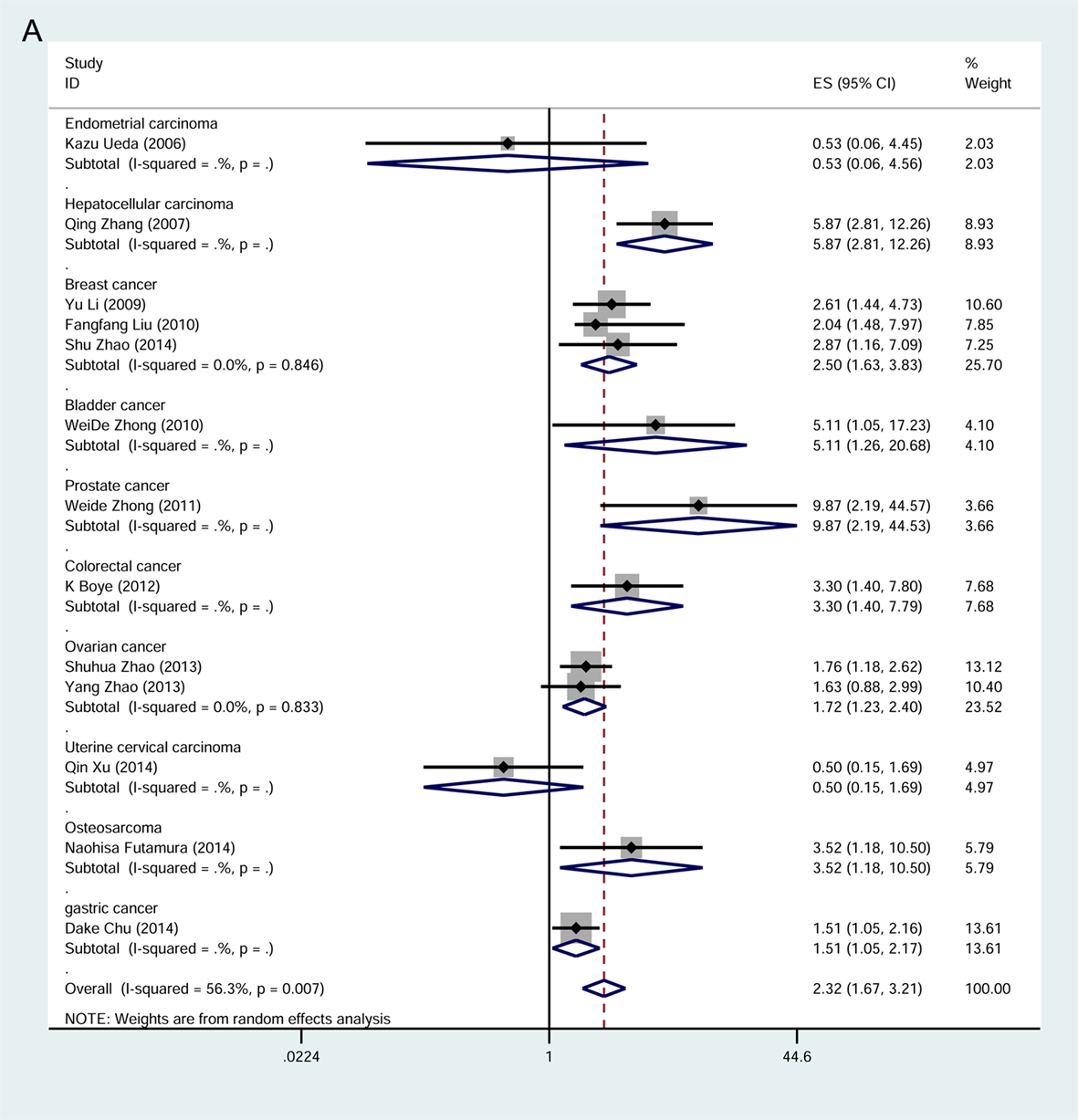


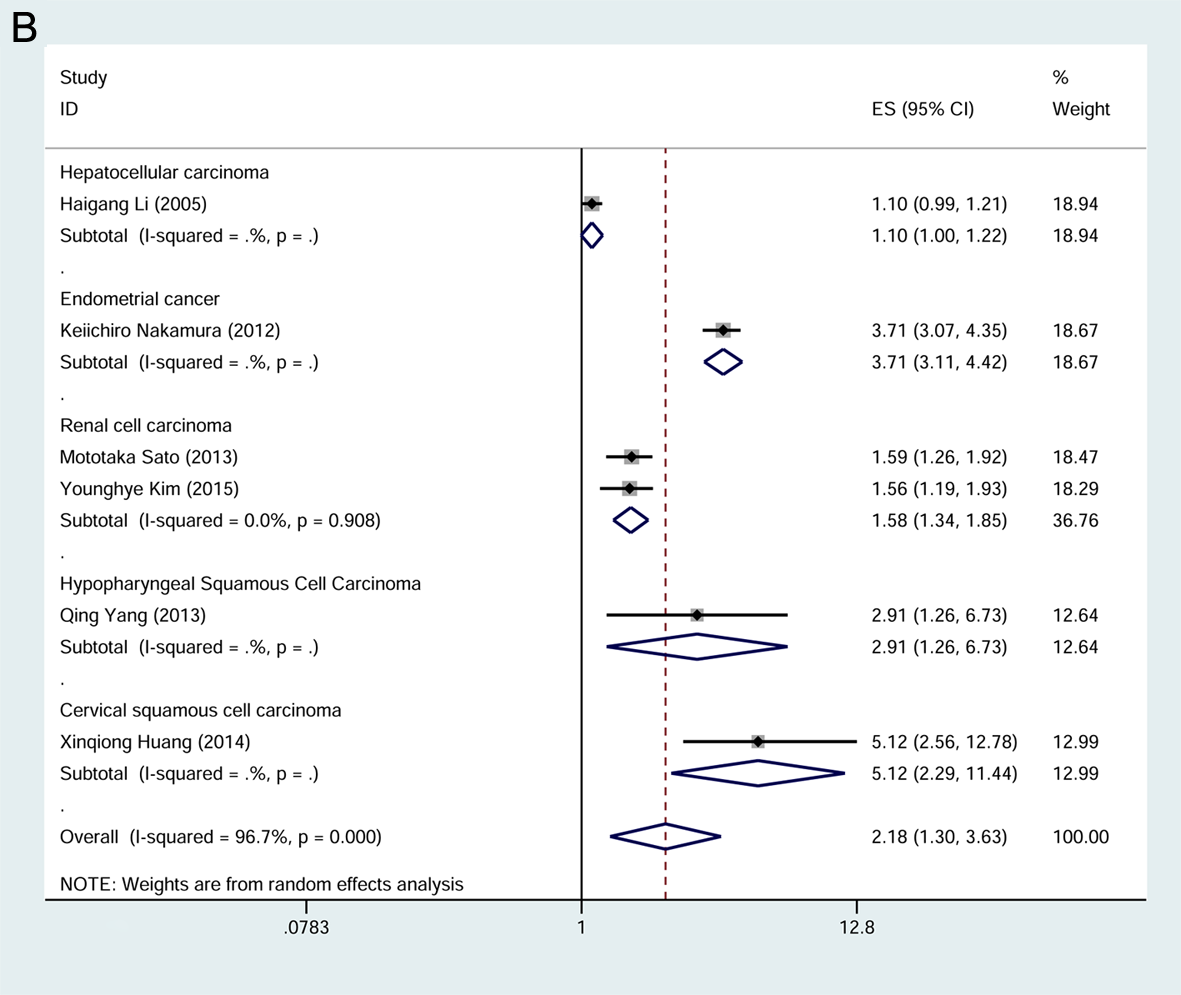


**Supplementary Figure 3.**

Sub-group analysis of the association between CD147/EMMPRIN over-expression and cancer patients. Panel represents sub-group analysis of CD147/EMMPRIN positive expression with DSS in multivariate models.


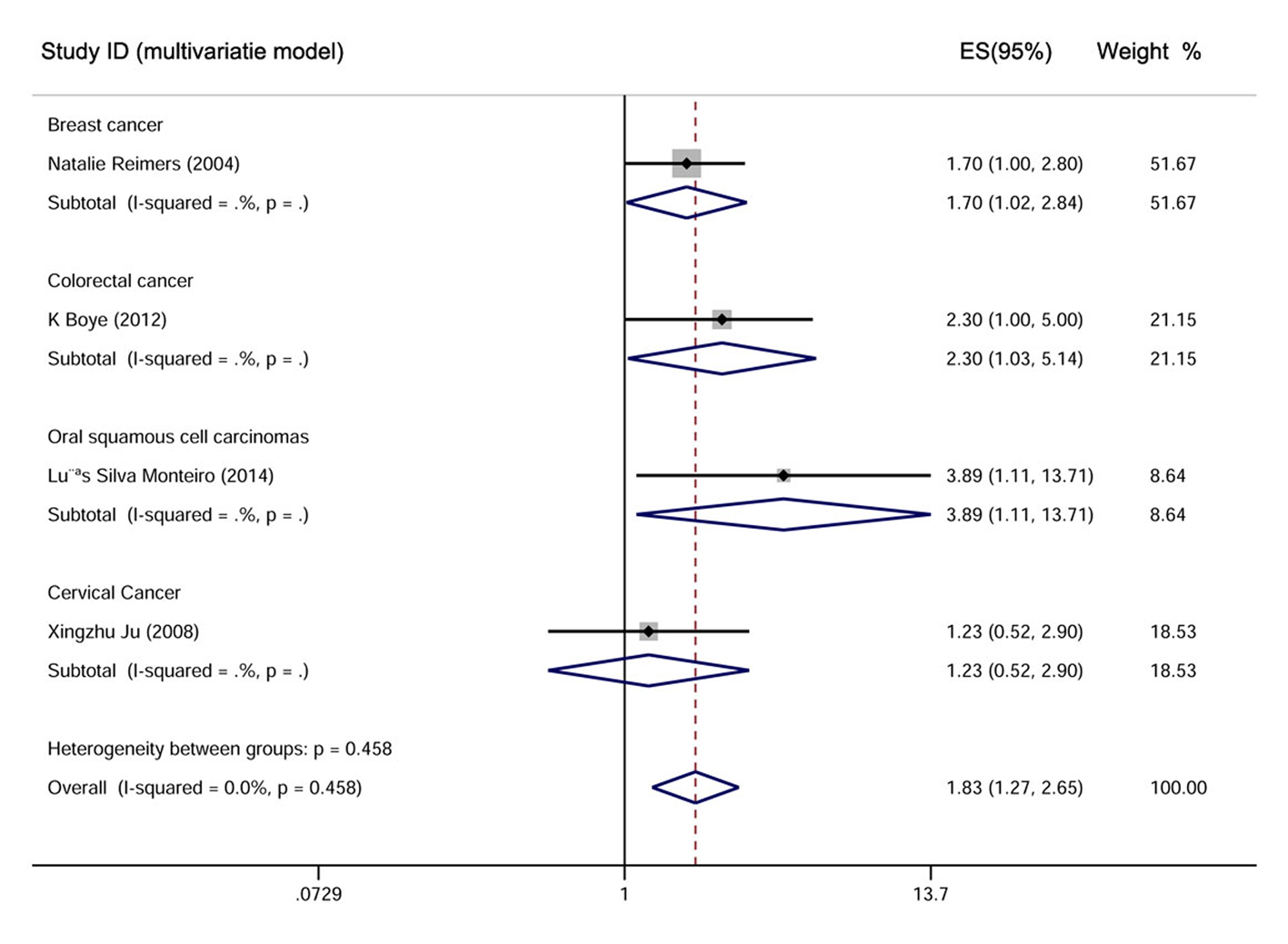


**Supplementary Figure 4.** Meta-regression for OS studies in multivariate(A1-A4), OS studies in univariate(B1-B3) and PFS studies in multivariate(C1-A4) with publication year, cancer type, sample size, and country as covariates. All covariates were entered into the meta-regression model simultaneously, and the covariates with the highest p values were omitted one at a time to identify sources of heterogeneity. We found cancer type may be the source of heterogeneity for OS studies in univariate (Coef.=0.093, p=0.042, B3) .


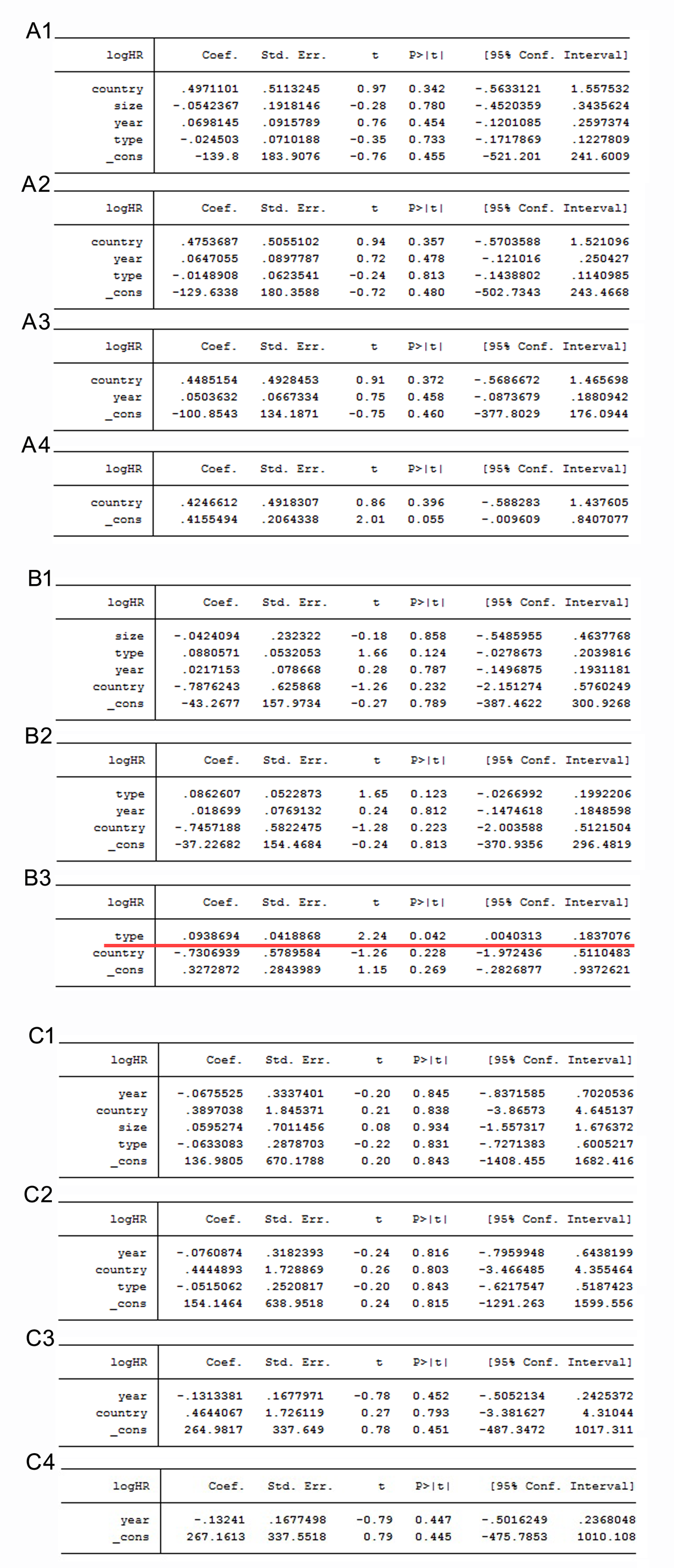

Supplement: Supplementary Information [file srep32804-s1.doc]
